# Image and clinical analysis of common carotid web: a case report

**DOI:** 10.1186/s12880-020-00536-6

**Published:** 2021-01-06

**Authors:** Man Gao, Jing Lei

**Affiliations:** Department of Neuroradioloy, Tianjin Huanhu Hospital, No. 6 Ji Zhao Road, Jin Nan District, Tianjin, 300350 China

**Keywords:** Carotid web, Common carotid artery, CTA, Stroke

## Abstract

**Background:**

A carotid web is a very rare vascular disease of the carotid artery, leading to thrombosis and ischemic stroke.

**Case presentation:**

A 65-year-old male patient was admitted due to left limb weakness. On arrival, he had moderate left hemiplegia, neglect, and sensory loss; the National Institutes of Health Stroke Scale score was 8. Computed tomography angiography (CTA) and magnetic resonance (MR) examination were performed to determine the cause of basal ganglia infarction. Thin-section axial CTA showed a membrane-like structure in the posterior wall of the right common carotid artery. The sagittal reconstruction image showed a membrane-like protrusion in the posterior wall of the right common carotid artery under the right carotid sinus. The MR axial T2 image showed a membrane-like high-signal protrusion into the carotid artery lumen, which was diagnosed as a right carotid web. The patient was treated with dual antihypertensive therapy by adjusting blood pressure, controlling brain edema, improving cerebral circulation, and nourishing the nerves.

**Conclusion:**

Careful comparison of axial thin-layer CTA and MR axial T2 images combined with sagittal reconstruction of CTA images can greatly improve the diagnostic rate of carotid web.

## Background

A carotid web is considered a rare fibromuscular dysplasia. It has received increasing attention for being a rare risk factor for ischemic stroke. It is a rare disease of the carotid artery, and its incidence is about 0.62% [1–3]. Because of the unclear or missed diagnosis of imaging diagnosis, clinical treatment is often not timely, so that patients can not get correct and timely treatment, thus aggravating the condition and affecting the quality of life. So its clinical and imaging definite diagnosis need further exploration.

## Case presentation

Standard care is performed, so ethical approval is not applicable in this study. Written informed consent was obtained from the patient.

A 65-year-old male patient was admitted to Tianjin Huanhu Hospital on October 20, 2018, due to left limb weakness accompanied by a headache and dizziness for 4 h. The computed tomography (CT) examination of the head indicated infarction of the right basal ganglia. He had a history of hypertension for 4 years, poor blood pressure control under ordinary circumstances, and a history of hyperlipidemia for more than 20 years. The physical examination revealed the following: blood pressure 177/110 mmHg (1 mmHg = 0.133 kPa), clear mind, unclear speech, a shallow nasolabial groove on the left side, left tongue extension, muscle strength of the left upper limb level 3, muscle strength of the left lower limb level 4, muscle strength of the right limb level 5, positive Babinski sign on the left side, incomplete finger nose test on the left side, and negative Kernig’s sign.

**Fig. 1:**
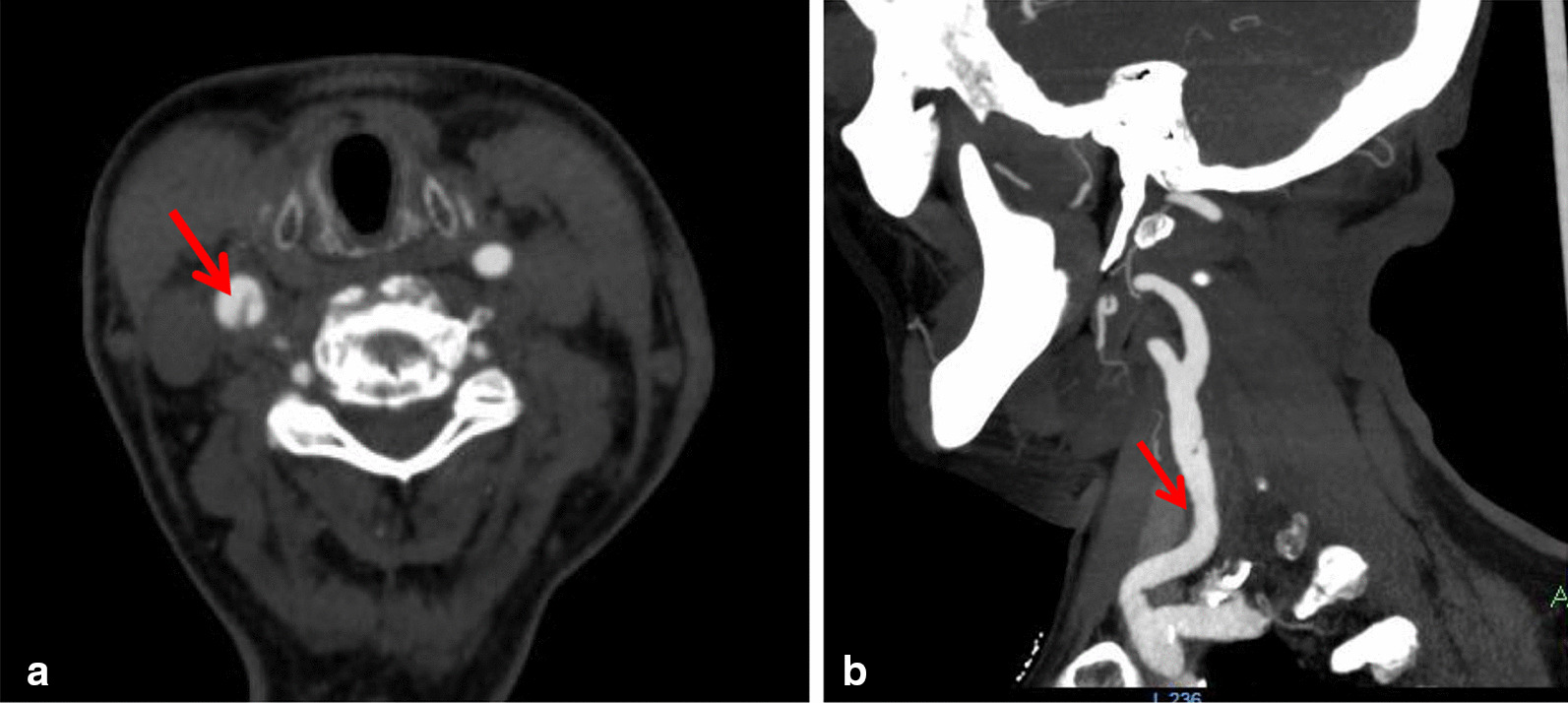
**a** CTA axial scan shows the filling defect of a thin-film contrast agent in the right carotid artery lumen, the main body of which is located deep into the lumen. **b** Sagittal CTA of the same patient showed that the web-like structure was located in the lumen at the end of the common carotid artery

**Fig. 2:**
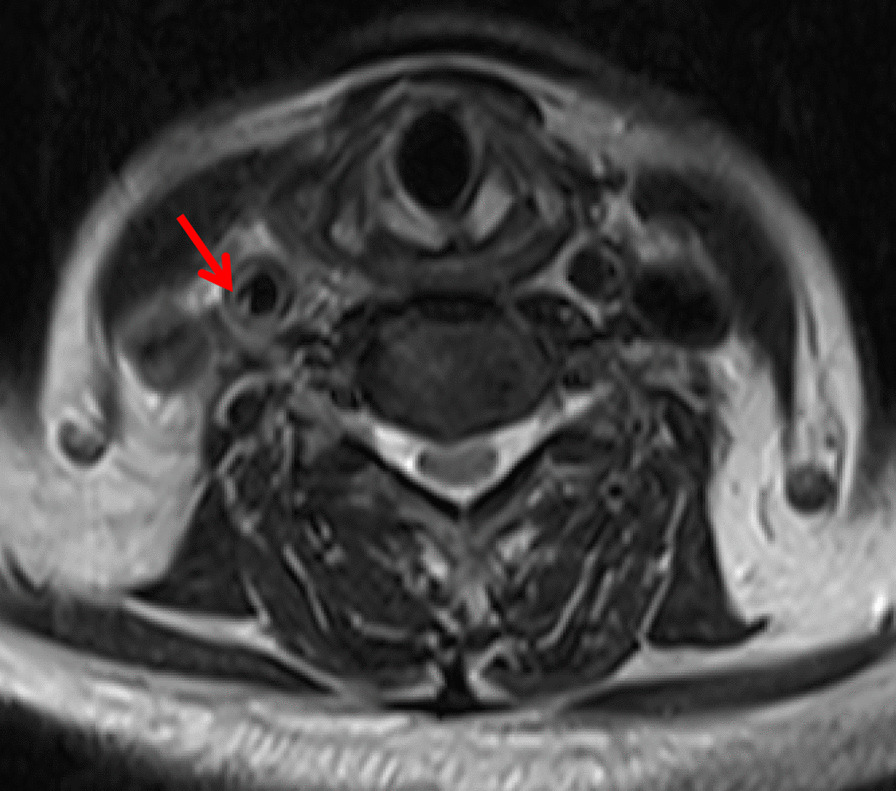
MR T2 image of the same patient showed that the thin-film-like slightly high signal was located in the lumen of the common carotid artery

Carotid CTA was performed on a 256-multidetector row CT scanner with 1-mm thick sections and was acquired from the aortic arch through the circle of Willis. Scanning parameters included the following: gantry rotation time, 0.5 s; pitch, 1; voltage, 120 kV; and 360 mAs CT scanner. GE 3.0 T MR scanner (General Electric Healthcare,) was used to acquire images using both conventional and research sequences. Thin-section axial CTA showed a membrane-like structure in the posterior wall of the right common carotid artery (Fig. 1a). The sagittal reconstruction image showed a membrane-like protrusion in the posterior wall of the right common carotid artery under the right carotid sinus (Fig. 1b). The MR axial T2 image showed a membrane-like high-signal protrusion into the carotid artery lumen (Fig. 2), which was diagnosed as a right carotid web. The patient was treated with aspirin 350 mg daily, clopidogrel 75 mg daily, enoxaparin sodium.

(Lovenox) 0.40 mL (40 mg) subcutaneously twice daily, and atorvastatin 40 mg daily. with reduction to single antiplatelet aspirin 350 mg daily after 2 months. At 8-month follow-up, the patient had residual speech difficulty and left-sided weakness.

## Discussion and conclusions

Currently, the etiology of a carotid web is unclear. It may be related to heredity, chronic vascular injury, hormone level, and trophoblastic vascular abnormality. In young women, it may be related to the use of oral contraceptives [4] because oral contraceptives can cause arterial intimal hyperplasia and the formation of a carotid web.

A carotid web is a special type of myofibrous dysplasia, with an angiogram different from the typical angiogram of myofibrous dysplasia [5]. Most of the angiograms show "bead-like" or focal "strip-like" stenosis, while a carotid web shows a membrane-like tissue protruding into the vascular cavity. Sajedi [6] et al. performed endarterectomy on two patients with a carotid web. The pathological results showed extensive hyperplasia of intimal muscle fibers with fibrosis and myxoid change. A review and retrospective analysis of the pathological results of 21 patients with a carotid web showed the abnormal variation of the intimal layer [7].

## Imaging examination of a carotid web and its characteristics

A carotid web can be found and diagnosed by magnetic resonance angiography (MRA), CTA, and digital subtraction angiography (DSA).

*DSA* DSA is the first examination method applied to the diagnosis of a carotid web. It is manifested as a linear filling defect in the vascular lumen and the retention of the contrast agent at the far end of the carotid web, leading to blood stasis, which is conducive to thrombosis. Although DSA is a safe and an invasive diagnostic method, the present case was not diagnosed by DSA because of dual antiplatelet therapy after CTA diagnosis.

*CTA* This technique can obtain detailed vascular morphological information and reconstruct in a short time. It has high sensitivity and specificity for the diagnosis of a carotid web. The CTA of the head and neck is often the first choice in clinical practice. It shows more specific manifestations of a carotid web: (1) a membrane-like structure in the artery lumen is seen in the axial position; (2) the posterior wall of the carotid sinus or the starting section of the internal carotid artery protrudes into the shelf-like filling defect in the lumen in the sagittal position; (3) in a few cases, thrombus can be seen on a carotid web. CTA can help distinguish other diseases, such as dissection, aneurysm, atherosclerosis, and so on. However, CTA also has some shortcomings, such as the inability to provide hemodynamic information. In addition, CTA is also associated with radiation-induced damage, which may cause renal damage, allergy, and so forth.

*MR* MR can provide varied information for the evaluation of a carotid web using different sequences and patterns. MR T2 shows a thin-film-like slightly high signal into the common carotid artery. Morphologically, the MRA and CTA of a carotid web are basically the same. However, MRA seems to be less sensitive and specific compared with CTA in the diagnosis of a carotid web. In one study, only intimal thickening was found on MRA in a case of carotid web confirmed by CTA [8]. The advantage of MRA is that it can provide more information about lesions and arterial wall and help differentiate and diagnose atherosclerosis and other diseases. Few studies explored the correlation between MRA and carotid web. Hence, the difference in the detection rate between different sequences still needs to be explored.

## Differential diagnosis of a carotid web

A focal atherosclerotic plaque (ASP) is characterized by an endometrial flap on axial CTA [9], and therefore it is easily misdiagnosed as an ASP [10]. The typical pathological features of an ASP are a lipid-rich necrotic core covered with a fibrous cap, multiple filling defects, and niches seen on the MIP map of CTA. The non-atherosclerotic and noninflammatory histopathological features of a carotid web are significantly different from those of an ASP; a membrane-like septal shadow is seen on a CTA image. Most patients with a carotid web have no vascular risk factors, differentiating it from atherosclerosis.

The direct imaging signs of carotid dissection are a double-lumen sign and an intimal flap. Trauma or spontaneity leads to the dissection of the carotid artery. Traumatic dissection is a serious complication of blunt trauma in the head and neck, which can lead to tearing of the intima of the vessel wall, formation of a hematoma, stenosis, and thrombosis in the lumen. Mild stressful actions (such as coughing, vomiting, exercise, or neck manipulation) do not directly cause spontaneous dissection, but they trigger spontaneous dissection in patients with the underlying arterial disease [11]. Spontaneous dissection is also the reason why cerebrovascular events are increasingly recognized in young and middle-aged patients. Further, 19.7% of patients with FMD have artery dissection [12], mostly involving the carotid artery. Therefore, it is necessary to identify such patients carefully in clinical practice.

## Treatment of a carotid web

Related studies have found that the recurrence rate of stroke in patients with a carotid web and ischemic stroke after antiplatelet therapy is about 30% [10, 13]. Therefore, monoclonal or dual antiplatelet therapy is not the best secondary prevention method. Anticoagulant therapy may be considered because the possible mechanism of thrombus formation is similar to that in the left atrial appendage.

In clinical treatment, the most commonly used carotid endarterectomy is the first operation method to prevent recurrent stroke in patients with a carotid web. The principle of preventing stroke by removing a carotid web is also obvious. A study on carotid endarterectomy in patients with a carotid web reported no recurrence of stroke [10]. The operation can effectively reduce the risk of stroke recurrence in such patients. Also, pathological tissues obtained in operation provides the conditions necessary for further studying the pathology and pathogenesis of a carotid web. However, carotid endarterectomy has many limitations, including high requirements of surgical technology, high risk of surgery, relatively high incidence of surgical complications, intolerance to surgery among some high-risk patients (old age, poor general condition, and so on), and so forth.

Some clinicians choose carotid stent placement for treating patients with ischemic stroke. In the study by Haussen [13], 16 patients were treated with carotid artery placement. The number of patients in a similar study was the largest, the average follow-up time was 4 months, and no recurrence of stroke was found. Further, the short-term prognosis was good [14]. Carotid artery stenting is a safe and effective method for treating a carotid web, with low perioperative complications and good short-term results. The time of carotid artery stent placement is shorter than that of carotid endarterectomy [15]. The present study had a small sample size. Also, no randomized controlled study involving the use of drug therapy or carotid endarterectomy is available at present. However, carotid artery stent therapy may have a great prospect with the development of neurointerventional technology and materials.

## Conclusion

Although carotid web is a rare disease, careful comparison of axial thin-layer CTA and MR axial T2 images, combined with CTA sagittal reconstruction, can greatly improve the diagnostic rate of carotid web, thus providing useful imaging information for clinical.

## Data Availability

The datasets used and/or analyzed during the current study are available from the corresponding author on reasonable request.

## Abbreviations

ASP: Atherosclerotic plaque
CT: Computed tomography
CTA: CT angiography
DSA: Digital subtraction angiography
MR: Magnetic resonance
MRA: Magnetic resonance angiography
